# The effect of using social pressure in cover letters to improve retention in a longitudinal health study: an embedded randomised controlled retention trial

**DOI:** 10.1186/s13063-017-2090-5

**Published:** 2017-07-20

**Authors:** Sarah Cotterill, Kelly Howells, Sarah Rhodes, Peter Bower

**Affiliations:** 1 Centre for Biostatistics, School of Health Sciences, University of Manchester, Manchester Academic Health Science Centre, Manchester, UK; 2 NIHR School for Primary Care Research, Manchester Academic Health Science Centre, University of Manchester, Manchester, UK

**Keywords:** Social pressure, General practice, Health behaviour, Primary care, Randomised controlled trial, Embedded, Health cohort study, Questionnaire design

## Abstract

**Background:**

Retention of participants in cohort studies is important for validity. One way to promote retention is by sending a persuasive cover letter with surveys. The study aimed to compare the effectiveness of a covering letter containing social pressure with a standard covering letter on retention in a health cohort study. Social pressure involves persuading people to behave in a certain way by the promise that their actions will be made know to others. We implemented a mild form of social pressure, where the recipient was told that information about whether they responded to the current survey would be noted by the research team and printed on future correspondence from the research team to the recipient.

**Methods:**

The design was an embedded randomised controlled retention trial, conducted between July 2015 and April 2016 in Salford, UK. Participants in the host health cohort study were eligible. They received either: (1) a covering letter with two consecutive surveys (sent six and twelve months after recruitment), containing a social pressure intervention; or (2) a matching letter without the social pressure text. The primary outcome was retention in the host study, defined as return of both surveys. Randomisation was computer-generated, with stratification by household size. Participants were blinded to group assignment. Researchers were blinded for outcome ascertainment.

**Results:**

Adults (*n* = 4447) aged over 65 years, with a long-term condition and enrolled in the host study, were randomly allocated to receive a social pressure covering letter (*n* = 2223) or control (*n* = 2224). All 4447 participants were included in the analysis. Both questionnaires were returned by 1577 participants (71%) sent the social pressure letters and 1511 (68%) sent control letters, a risk difference of 3 percentage points (adjusted odds ratio = 1.16 (95% confidence interval = 1.02–1.33)).

**Conclusion:**

A mild form of social pressure made a small but significant improvement in retention of older adults in a health cohort study. Investigation of social pressure across other research contexts and stronger social pressure messages is warranted.

**Trial registration:**

The host cohort study, the Comprehensive Longitudinal Assessment of Salford Integrated Care (CLASSIC) study is associated with the CLASSIC PROTECTs trial, which is registered on the ISRCTN registry. Trial registration number: ISRCTN12286422. Date of registration 19 June 2014.

**Electronic supplementary material:**

The online version of this article (doi:10.1186/s13063-017-2090-5) contains supplementary material, which is available to authorized users.

## Background

The recruitment and retention of participants to health research presents challenges for researchers. A randomised controlled trial (RCT) that is embedded within a host research study, testing one or more alternative research designs, is a suitable way to examine which aspects of research design are most effective: such an approach has been suggested for improving response to research questionnaires [1, 2] and enhancing trial recruitment [3–5]. Embedded trials can also be useful in identifying the most effective methods of retention [4]. We define retention as the process of keeping a participant involved in a research study after they have agreed to take part.

Postal questionnaires are widely used as a method of data collection in health research and are a cost-effective option compared with telephone and email [6]. Non-response to questionnaires reduces the available sample size, decreases the precision of parameter estimates and can introduce non-response bias [1, 7]. There is a long history of research on methods to improve survey response [8, 9]. There is strong evidence that financial rewards (and to a lesser extent financial incentives) can improve questionnaire response rates [10], even when the amounts are small [11], and that shorter questionnaires [12–14] and reminders [13–15] are often effective. Response is also conditional on study-related factors including the organisation sending the letter and how the recipient regards them [8, 9]. Of particular relevance to this study, personalisation of the cover letter can improve response, such as adding a wet-ink signature [16, 17] or addressing the respondent by name [16, 18]. Personalisation is not always effective [5, 18]. This variation in effect is influenced by the competing effects of personalisation, which seeks to build a connection with the recipient, and anonymity, which tries to achieve the opposite, creating a ‘two-edged sword’, particularly in surveys requesting sensitive information [8].

One variant of personalisation is social pressure, which involves persuading people to behave in a certain way by the promise that their actions will be made public [19]. It arises because we are all eager to be seen in a positive light by those around us. Public praise and shame make a behaviour more salient and can encourage citizens to comply with a request [20]. Pride arising from acclaim by peers can motivate people to persevere in carrying out actions, despite obstacles [21]. In economics, ‘image motivation’ describes how citizens seeking social approval may choose to exhibit qualities that they think are widely regarded as good. Research indicates that people are more likely to act in a prosocial way in a public space than in a private space [22] and that charitable donors enjoy the feeling of ‘prestige’ they experience when their donations are publicised [23]. In social sciences, ‘social pressure’ can be effective in raising voter turnout in elections [19, 24] and in encouraging charitable donations [25, 26]. Social pressure conveys to the participant that information about their behaviour will be noticed. It can be done in a way that either engenders pride or shame, although in health research the utilisation of shame is likely neither desirable nor ethical. Social pressure can also be regarded as a social incentive, which informs the recipient that a verbal or non-verbal reward will be delivered if there is progress in performing the behaviour [27].

Social pressure is unlikely to be effective among those who are very committed to the behaviour or those who are not at all interested: it is particularly suited to those who have undertaken the behaviour before or are already thinking about the behaviour, but in need of persuasion or reminder [24]. This makes it particularly suitable for a longitudinal health survey, where the participants have already completed an initial questionnaire and indicated willingness for future participation.

We undertook an RCT to test the application of social pressure to questionnaire retention. We implemented a mild form of social pressure, letting participants know that their behaviour was being monitored [28], which we thought would be more appropriate in this context than stronger versions of social pressure, which promise to advertise the behaviour in a public place. The objective of the embedded trial was to test the effect of notifying survey participants that their responses are being noticed compared to a simple letter control, on response to a longitudinal health survey. We follow the CONSORT trial reporting guidelines [29–31], including the extension for embedded recruitment trials [4]. The protocol is available from the lead author.

## Methods

### Trial design

We implemented a parallel group RCT which was embedded in a longitudinal health survey. Participants were randomly allocated 1:1 to receive standard covering letters (control condition) or the same covering letter with additional wording to convey that participant responses were being noticed. The letters were sent to accompany two consecutive cohort questionnaires, at six and 12 months after baseline; each letter was posted simultaneously to all participants in both experimental groups. The primary outcome was response to both surveys, to test for the effect on retention. The trial was conducted between July 2015 and April 2016 in Salford, UK.

### Host study

The Salford Integrated Care Programme (SICP) is a large-scale integrated-care programme to improve care for older people with long-term conditions and social-care needs in Salford, a city in northwest England which has high levels of deprivation and long-term illness. The Comprehensive Longitudinal Assessment of Salford Integrated Care (CLASSIC) (the host study) is a cohort study, designed to provide a rigorous test of the ability of the SICP to deliver improved care for older people. The CLASSIC cohort was recruited and followed over time, to assess the overall impact of SICP, while subgroups of the cohort were used to evaluate different SICP interventions.

All general practices were approached to participate in the CLASSIC study and 33 out of a possible 47 were recruited. Inclusion criteria were patients aged over 65 years who were on practice registers for at least one of the following long-term conditions: atrial fibrillation; high blood pressure; coronary heart disease (CHD); heart failure; stroke, chronic obstructive pulmonary disease (COPD); asthma; chronic kidney disease (CKD); diabetes; epilepsy; psoriasis; rheumatoid arthritis’ and osteoarthritis. CLASSIC employed an innovative recruitment approach using FARSITE, which enables researchers to search anonymised GP health records. FARSITE was used to manage and create lists of eligible patients and then each practice was asked to identify patients meeting the exclusion criteria (patients in palliative care and those with conditions which reduce capacity to consent and participate). Once approved by GPs, a link with a third-party service enabled the invitation letters to be mailed remotely. Practices did not receive incentives to take part but did receive support costs to reimburse their time.

### Participants in the host study

**Fig. 1:**
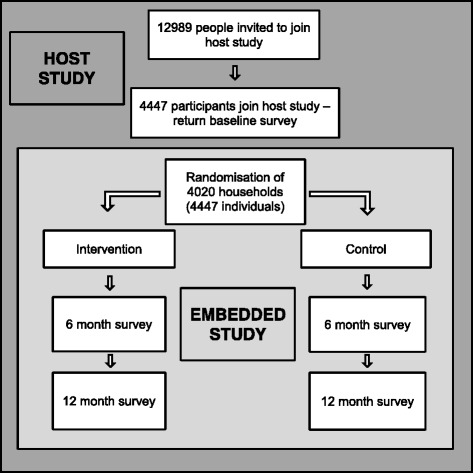
*Flow chart* of participants in the host study and embedded study

The target population for the host study were people aged over 65 years with a long-term condition. The host study recruited 4447 people, who consented to receive four questionnaires on a six-monthly basis. This was out of 12,989 eligible patients who were sent an initial baseline questionnaire in January 2015. The eligibility conditions for the embedded study were the same as the host study and all host study participants were included. The flow of participants through the host and embedded studies is illustrated in Fig. 1.

Participants were sent four postal surveys, at baseline and then every six months thereafter. The surveys in which this RCT was embedded were the second and third surveys, which were sent at six and 12 months after baseline. The surveys were similar to one another and contained brief measures of service experience, health and care outcomes and service utilisation. These measures were linked to routine data from electronic records on clinical parameters, medication use and interactions with NHS and social care services. Maximising retention across all four surveys was crucial for the validity of the host study. The second questionnaire was sent in July 2015 to those who had completed the first questionnaire. The third questionnaire was sent in January 2016 to everyone in the cohort, except those who actively withdrew or died. At each stage, participants who did not return a questionnaire were sent a second copy with a reminder letter three weeks later. Participants in both groups were offered an incentive of a £10 voucher for completion of the first (baseline) questionnaire and £5 for completion of the third questionnaire, but no incentive was given for completion of the second questionnaire.

### Intervention

All participants were sent a covering letter and a copy of the CLASSIC questionnaire. The content of the intervention and control letters was similar (Additional file 1) and the intervention letter with the first questionnaire included the following additional text. The wording of the intervention was designed to exert social pressure by letting the recipient know that their previous response had been noted and that future responses would be noted by the researcher and communicated to the recipient at the time of the subsequent questionnaire:
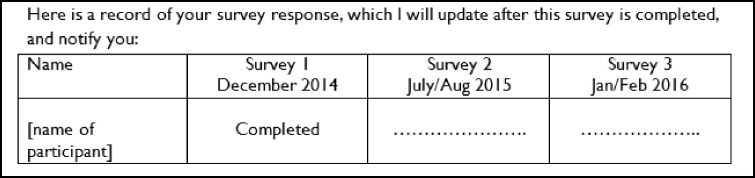


The intervention letter with the second questionnaire included the following additional text, which was designed to exert further social pressure by letting the recipient know that their previous two responses had been noted and that future responses would be communicated to the recipient at the time of the subsequent questionnaire:
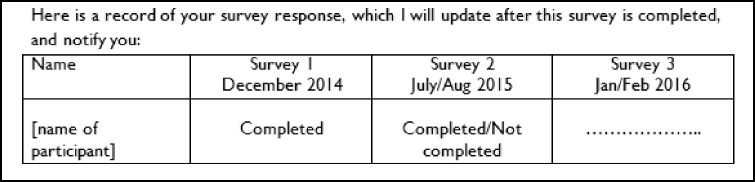


Participants in the control group received the standard covering letter, similar to the intervention group, but without the additional text. Both intervention and control groups received the same encouragement from the researchers on the host study to return completed questionnaires, including financial incentives and follow-up phone calls.

### Outcomes

The objective of the intervention was to improve retention in a cohort study, so we focus the primary outcome on completeness of response. We were interested in ‘unit’ response, defined as whether the respondent returns the survey, rather than ‘item’ response, defined as whether the respondent completes particular questions on the survey. The primary outcome for the embedded trial was retention in the cohort, measured by the return of both completed questionnaires. The definition of a returned questionnaire was that adopted by the host study: any questionnaire with at least one completed scale. Secondary outcomes were: return of a completed first questionnaire; return of a completed second questionnaire; and return of either questionnaire.

The original protocol was concerned with the first questionnaire and specified the primary outcome as unit response to the first questionnaire. The primary outcome was changed to ‘unit response to both questionnaires’ on the recommendation of the chief investigator of the host cohort study prior to him having access to the response data to the second questionnaire. This was viewed by him as the most important endpoint with regards to retention in a cohort study.

### Sample size

A conventional sample size calculation was not appropriate, as the sample size was determined by the size of the host study (the 4447 individuals who had already been recruited to the CLASSIC cohort and returned a baseline questionnaire). We nevertheless present some estimates of power given likely effects. The cohort included 380 pairs of individuals living in the same household, most of whom were couples. We expected that individuals in the same household were likely to have sight of each other’s letters and we wanted to avoid contamination between different experimental conditions, so we ensured that all members of a household were assigned to the same intervention. The available sample of 4447 individuals provides 80% power to detect a difference of around 4.5 percentage points between a control group response rate of 70% and a treatment group response rate of 74.5%, which equates to an odds ratio (OR) of around 1.2 (two-sided, *p* > 0.05), which is similar to the effect of a similar letter on voter turnout [28]. We have not accounted for clustering by household in the sample size calculation, because the effect was anticipated to be small, but we have taken account of it in the analysis.

### Randomisation

Randomisation was undertaken at the household level and stratified by household size to ensure that the multiple occupancy households were evenly distributed between the two groups and to keep the groups approximately equal in size. A random number variable was generated, uniformly distributed between 0 and 1. The single-person households were randomly assigned to treatment and control groups, using blocking to achieve groups of equal size. This was repeated for the two- and three-person households. Randomisation was undertaken using Stata 14 [32].

### Blinding

Participants were blinded to their participation in the embedded study. They were not informed that other households were being sent differently worded letters: we expected that their actions might change if they knew what others receive. This approach was acceptable to the research ethics committee. Randomisation was performed after recruitment to the host study, ensuring allocation concealment. The research team were not blinded to the intervention, but had minimal contact with trial participants. Researchers were blinded to group assignment during outcome ascertainment.

### Statistical analysis

Data management followed the existing procedures for the host study. Possible dispositions [33] were: returned questionnaire; died; withdrew (participants were advised that the return of a blank questionnaire would constitute withdrawal); not returned; and non-contact. For the primary intention-to-treat (ITT) analysis, the disposition is categorised as returned or not returned (which includes died, withdrew, not returned and non-contact). We present summary descriptive statistics of participants by group. We estimate the effect of group allocation on unit response, using logistic regression. To control for multiple participants per household, bootstrapping with adjustment for clustering was used, with 1000 replications. To adjust for any baseline imbalance, covariates for age (over 75 years), poor quality of life and male gender, living alone and having no qualifications were included in the model. All analyses were carried out in Stata 14 [32]. The main analyses were performed on an ITT basis, including all randomised participants, whether or not they received a questionnaire. We conducted a sensitivity analysis where participants with a missing address were excluded.

## Results

**Fig. 2:**
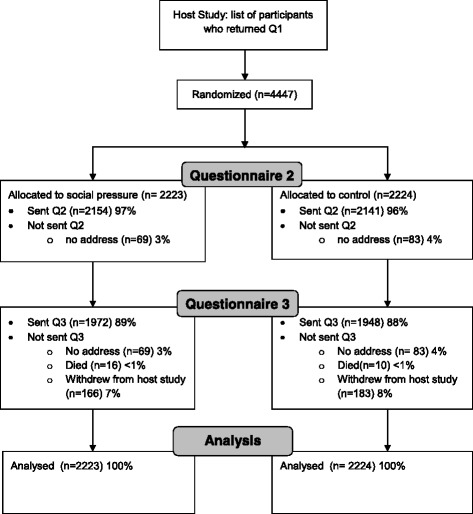
Consort *flow diagram*

The first questionnaire was posted in July to August 2015 and the second in January to February 2016. The flow of participants through the trial is shown in Fig. 2. All 4447 participants who had responded to an earlier questionnaire were randomised to receive the social pressure letter (*n* = 2223) or a control letter (*n* = 2224). Questionnaires were not sent to people without an address on record and those who were known to have died or withdrawn from the host study. No participants were lost to follow-up in the embedded retention trial: all 4447 participants provided an outcome measure simply by returning or not returning the questionnaire (Fig. 2).

The baseline demographic characteristics and health status of trial participants are presented in Table 1. The mean age of the trial population was 75 years, just over half were women (54%) and they were largely of white British ethnicity (95%). Half of the sample had no qualifications and one-fifth needed help with reading materials. All the participants had long-term health conditions; on average, each person reported five health conditions [34]. Quality of life was rated as good or very good by 71% of participants; 52% said they were satisfied with their health, measured by WHOQOL [35]. Characteristics at enrolment were balanced between the two groups.

**Table 1:** Participant characteristics and health status at baseline, by group

	Social pressure intervention *N* = 2223	Control *N* = 2224
Gender (*N* = 4329)		
Male n (%) Female n (%)	993 (46) 1170 (54)	1027 (47) 1139 (53)
Age (*N* = 4090)		
Mean (SD) Median (Min,Max)	74.9 (6.9) 74 (65,97)	74.7 (6.7) 74 (65,98)
Number of study participants in household (*N* = 4447)		
1 n (%) 2 n (%) 3 n (%)	1821 (82) 402 (18) 0 (0)	1821 (82) 400 (18) 3 (0.1)
Current home status (*N* = 4316)		
Lives alone n (%) Does not live alone n (%)	828 (38) 1334 (62)	745 (35) 1409 (65)
Ethnicity (*N* = 4315)		
White British n (%) Other n (%)	2044 (95) 115 (5)	2070 (96) 86 (4)
Education (*N* = 4049)		
No qualifications n (%) At least 1 O-Level or equivalent n (%)	1012 (50) 1003 (50)	991 (49) 1043 (51)
How often help is needed with reading materials (*N* = 4220)		
Never/Rarely n (%) Sometimes/Often/Always n (%)	1699 (80) 415 (20)	1693 (80) 413 (20)
Quality of life (from WHOQOL) (*N* = 4257)		
Very poor/poor/neutral n (%) Good/very good n (%)	611 (29) 1518 (71)	612 (29) 1516 (71)
Satisfaction with health (from WHOQOL) (*N* = 4249)		
Very dissatisfied/dissatisfied/neutral n (%) Satisfied/very satisfied n (%)	1011 (48) 1110 (52)	1010 (47) 1118 (53)
Number of long-term conditions (using BAYLISS) (*N* = 4329)		
Mean (SD) Median (Min,Max)	5.4 (3.1) 5 (0,19)	5.4 (3.2) 5 (0,21)

All 4447 randomised participants were included in the primary ITT analysis. The outcome was a binary measure of whether a questionnaire was returned. We are not aware of any participants receiving a different intervention than the one they were allocated.

A summary of all observed outcomes and treatment effect estimates is given in Table 2. The primary outcome is return of questionnaires 2 and 3. Both questionnaires were returned by 1577 participants (71%) who were assigned to the social pressure letter and 1511 (68%) assigned to the control letter, an increase of 3 percentage points. The OR (95% confidence interval [CI]) is 1.15 (1.01–1.31). This effect is sustained with an or (95% CI) of 1.16 (1.02–1.33) after adjusting for clustering within households and factors thought to affect response: age; quality of life; gender; living alone; and education. All the secondary outcomes, questionnaire return at six months, questionnaire return at 12 months and return of at least one questionnaire, showed no statistically significant difference between the two groups, although for all outcomes, the response was higher in the social pressure group than in the control group.

**Table 2:** Results: questionnaire return by experimental condition, using logistic regression adjusting for household and baseline characteristics and clustering

Outcome		Intervention *N* = 2223 n (%)	Control *N* = 2224 n (%)	Risk difference (unadjusted) (%)	Odds ratio (95% CI) Intervention vs. control	*p* value
Primary	Both questionnaires returned	1577 (71)	1511 (68)	3	1.15 (1.01–1.31)	0.030
					1.15^a^ (1.01–1.31)	0.036
					1.16^b^ (1.02–1.33)	0.021
Secondary	At least one of questionnaires returned	1809 (81)	1787 (80)	1	1.07 (0.92–1.24)	0.385
					1.07^a^ (0.90–1.28)	0.463
					1.07^b^ (0.92–1.25)	0.348
	Six-month questionnaire returned	1739 (78)	1701 (76)	2	1.10 (0.95–1.27)	0.165
					1.10^a^ (0.94–1.30)	0.233
					1.11^b^ (0.96–1.28)	0.149
	12-month questionnaire returned	1647 (74)	1597 (72)	2	1.12 (0.98–1.28)	0.087
					1.12^a^ (0.98–1.28)	0.086
					1.14^b^ (0.99–1.29)	0.062

^a^Adjusted for multiple participants per household

^b^Adjusted for age, quality of life, gender, living alone and education

In a sensitivity analysis, we repeated the analysis excluding 152 randomised participants who were not sent either questionnaire because their address was unavailable. Excluding these participants from the unadjusted analysis of the primary outcome leads to an OR (95% CI) of 1.14 (1.00–1.30) so the conclusions are robust regardless of whether we exclude these participants. It is usual practice to report the intracluster correlation coefficient (ICC) in cluster trials, but in this trial there is so little clustering that the ICC would be uninformative: 3642 clusters (90.1%) were single-person households and only 402 clusters (9.9%) were households of two or more persons.

## Discussion

This evidence suggests that the addition of a simple social pressure intervention to a covering letter may lead to a modest increase in the number of participants returning more than one questionnaire. Use of social pressure interventions of this kind can aid retention in health cohort studies and the effect is similar to that found for other methods of personalising covering letters [18]. The response to the intervention was similar at both time points, with no sign of a diminished effect. It would be feasible to deliver social pressure to a cohort over a longer period, but we have no evidence on whether it would continue to be effective. It is plausible that the effect might wane, particularly with repeated non-responders receiving letters confirming their history of non-response. The only additional cost to run the study was £380 for the postal company to assign the correct letter to each respondent. The intervention requires minimal cost and effort and even modest effects of the size seen here may justify their implementation. Modest interventions, such as social pressure, are never likely to have large effects, but a combination of best practice modifications like this may combine to show more substantive benefit [36]. Fifteen years on, we can echo Don Dilman’s call for more research on how different approaches can complement one another and how they vary by population and topic [9].

The study population were older adults, with multiple long-term conditions. This older cohort, in common with people of this age group across the UK [37], had a relatively low level of formal education. Further investigation of the intervention in different situations such as longer-term cohort studies, cohorts with different patient groups, other age groups and in randomised trials is warranted.

The intervention implemented here was a very mild form of social pressure, based on highlighting to the recipient that their actions are being noticed by the sender of the questionnaire. The modest success of this mild form may suggest that investigation of interventions using stronger social pressure messages is warranted. Stronger social pressure in this context could include inducing pride by offering in advance to publicise the details of those who respond to the questionnaire, for example by naming consenting responders in a study newsletter. There is likely to be an optimal amount of pressure in this context: very strong social pressure used in other settings, such as shaming people for non-response, would be counter-productive and unacceptable in a health context. The acceptability of social pressure interventions will likely vary according to population type, questionnaire topic and country regulations, for example, social pressure may fall foul of the ‘common rule’ (45 CFR 46) in the United States [38].

The benefits of undertaking an embedded trial of this type are that the financial cost and time effort were minimal over and above those of the host study. Some additional work was involved in allocating people to different interventions and in sending out alternate versions of the letter, tailored to group allocation and nature of their response. No additional recruitment, consent or data collection were required, making effective use of participants’ time. This embedded trial methodology provides a valuable opportunity for rapid investigation of novel retention interventions, 12 months from first idea to results in this example. There is minimal ascertainment bias as we got ethical approval that participants were unaware of the study.

The study design presents some minor challenges, the main one being the constraints on sample size, which, unlike embedded recruitment trials, cannot exceed the sample size of the host study. Researchers on the embedded trial are reliant on the host study for data collection and management, and may have little control over the timing and methods. The design of the host study may introduce analysis issues, such as in this example, the clustering of participants in households. These all proved to be minor limitations, compared with the benefits.

## Conclusions

The addition of a simple social pressure intervention to a covering letter accompanying a health cohort questionnaire leads to a modest increase in the number of participants returning more than one questionnaire. The intervention requires minimal cost and effort, and consequently even modest effects of the size seen here may justify their implementation. An embedded RCT is a practical and efficient method of conducting research on the effect of behaviour change interventions on retention.

## Additional file

Additional file 1: Full text of the covering letters sent with the CLASSIC questionnaires. (PDF 66 kb)

## Abbreviations

CHD: Coronary heart disease
CI: Confidence interval
CLASSIC: Comprehensive Longitudinal Assessment of Salford Integrated Care
CONSORT: Consolidated Standards of Reporting Trials
COPD: Chronic obstructive pulmonary disease
FARSITE: Feasibility Assessment and Recruitment System for Improving Trial Efficiency
GP: General practitioner
ICC: Intracluster correlation coefficient
ITT: Intention to treat
RCT: Randomised controlled trial
WHOQOL: The World Health Organization’s quality of life assessment tool

## References

[1] McColl E, Jacoby A, Thomas L, Soutter J, Bamford C (2002). Design and use of questionnaires: a review of best practice applicable to surveys of health service staff and patients. Health Technol Assess.

[2] Nakash RA, Hutton JL, Jørstad-Stein EC, Gates S, Lamb SE (2006). Maximising response to postal questionnaires – A systematic review of randomised trials in health research. BMC Med Res Methodol.

[3] Rick J, Graffy J, Knapp P, Small N, Collier DJ, Eldridge S (2014). Systematic techniques for assisting recruitment to trials (START): study protocol for embedded, randomized controlled trials. Trials.

[4] Madurasinghe VW, Sandra Eldridge on behalf of MRC START Group (2016). Gordon Forbes on behalf of the START Expert Consensus Group: Guidelines for reporting embedded recruitment trials. Trials.

[5] Brueton VC, Tierney J, Stenning S, Harding S, Meredith S, Nazareth I (2013). Strategies to improve retention in randomised trials. Cochrane Database Syst Rev.

[6] Sinclair M, O’Toole J, Malawaraarachchi M, Leder K (2012). Comparison of response rates and cost-effectiveness for a community-based survey: postal, internet and telephone modes with generic or personalised recruitment approaches. BMC Med Res Methodol.

[7] Moser CA, Kalton G (1985). Survey Methods in Social Investigation.

[8] Linsky AS (1975). Stimulating responses to mailed questionnaires: a review. Public Opin Q.

[9] Dillman DA (1991). The design and administration of mail surveys. Annu Rev Sociol.

[10] Gates S, Williams MA, Withers E, Williamson E, Mt-Isa S, Lamb SE (2009). Does a monetary incentive improve the response to a postal questionnaire in a randomised controlled trial? The MINT incentive study. Trials.

[11] Edwards P, Cooper R, Roberts I, Frost C (2005). Meta-analysis of randomised trials of monetary incentives and response to mailed questionnaires. J Epidemiol Community Health.

[12] Edwards P, Roberts I, Sandercock P, Frost C (2004). Follow-up by mail in clinical trials: does questionnaire length matter?. Control Clin Trials.

[13] Glidewell L, Thomas R, MacLennan G, Bonetti D, Johnston M, Eccles MP (2012). Do incentives, reminders or reduced burden improve healthcare professional response rates in postal questionnaires? two randomised controlled trials. BMC Health Serv Res.

[14] Sahlqvist S, Song Y, Bull F, Adams E, Preston J, Ogilvie D (2011). Effect of questionnaire length, personalisation and reminder type on response rate to a complex postal survey: randomised controlled trial. BMC Med Res Methodol.

[15] Bauman A, Phongsavan P, Cowle A, Banks E, Jorm L, Rogers K (2016). Maximising follow-up participation rates in a large scale 45 and Up Study in Australia. Emerg Themes Epidemiol.

[16] Scott P, Edwards P (2006). Personally addressed hand-signed letters increase questionnaire response: a meta-analysis of randomised controlled trials. BMC Health Serv Res.

[17] Renfroe EG, Heywood G, Foreman L, Schron E, Powell J, Baessler C (2002). The end-of-study patient survey: methods influencing response rate in the AVID Trial. Control Clin Trials.

[18] Edwards PJ, Roberts I, Clarke MJ, DiGuiseppi C, Wentz R, Kwan I (2009). Methods to increase response to postal and electronic questionnaires. Cochrane Database Syst Rev.

[19] Green D, Gerber A (2010). Introduction to social pressure and voting: new experimental evidence. Polit Behav.

[20] Bénabou R, Tirole J (2006). Incentives and prosocial behavior. Am Econ Rev.

[21] Williams LA, DeSteno D (2008). Pride and perseverance: The motivational role of pride. J Pers Soc Psychol.

[22] Ariely D, Bracha A, Meier S (2009). Doing good or doing well? Image motivation and monetary incentives in behaving prosocially. Am Econ Rev.

[23] Harbaugh WT (1998). What do donations buy? A model of philanthropy based on prestige and warm glow. J Public Econ.

[24] Panagopoulos C (2010). Affect, social pressure and prosocial motivation: field experimental evidence of the mobilizing effects of pride, shame and publicizing voting behavior. Polit Behav.

[25] Cotterill S, John P, Richardson L (2013). The impact of a pledge request and the promise of publicity: a randomized controlled trial of charitable donations. Soc Sci Q.

[26] Cotterill, S. 2017 The influence of population characteristics on household response to a charity book collection based on pledges and social pressure. International Journal of Nonprofit and Voluntary Sector Marketing, 22.e1572. doi:10.1002/nvsm.1572.

[27] Michie S, Richardson M, Johnston M, Abraham C, Francis J, Hardeman W (2013). The behavior change technique taxonomy (v1) of 93 hierarchically clustered techniques: building an international consensus for the reporting of behavior change interventions. Ann Behav Med.

[28] Gerber AS, Green DP, Larimer CW (2008). Social pressure and voter turnout: Evidence from a large-scale field experiment. Am Polit Sci Rev.

[29] Schulz KF, Altman DG, Moher D (2010). CONSORT 2010 statement: updated guidelines for reporting parallel group randomized trials. Ann Intern Med.

[30] Hopewell S, Clarke M, Moher D, Wager E, Middleton P, Altman DG (2008). CONSORT for reporting randomised trials in journal and conference abstracts. Lancet.

[31] Hopewell S, Clarke M, Moher D, Wager E, Middleton P, Altman DG (2008). CONSORT for Reporting Randomized Controlled Trials in Journal and Conference Abstracts: Explanation and Elaboration. PLoS Med.

[32] StataCorp (2015). Stata Statistical Software: Release 14.

[33] The American Association for Public Opinion Research. 2016. Standard Definitions: Final Dispositions of Case Codes and Outcome Rates for Surveys. 9th edition. AAPOR. http://www.aapor.org/Standards-Ethics/Standard-Definitions-(1).aspx.

[34] Bayliss EA, Ellis JL, Steiner JF (2009). Seniors’ self-reported multimorbidity captured biopsychosocial factors not incorporated into two other data-based morbidity measures. J Clin Epidemiol.

[35] Skevington SM, Lotfy M, O’Connell KA (2004). The World Health Organization’s WHOQOL-BREF quality of life assessment: psychometric properties and results of the international field trial. A report from the WHOQOL group. Qual Life Res.

[36] Treweek S, Altman DG, Bower P, Campbell M, Chalmers I, Cotton S (2015). Making randomised trials more efficient: report of the first meeting to discuss the Trial Forge platform. Trials.

[37] Highest levels of qualification across England and Wales infographic. http://webarchive.nationalarchives.gov.uk/20160105160709/. Accessed 14 July 2017.

[38] Policy for Protection of Human Research Subjects, Revised January 15 2009 http://www.hhs.gov/ohrp/regulations-and-policy/regulations/45-cfr-46/.

