# Supplementary material for: The effect of using social pressure in cover letters to improve retention in a longitudinal health study: an embedded randomised controlled retention trial
Source: Trials. 2017 Jul 20;18:341. doi: 10.1186/s13063-017-2090-5 (PMC5520383; doi:10.1186/s13063-017-2090-5)
Supplement: Additional file 1: — Full text of the covering letters sent with the CLASSIC questionnaires. (PDF 66 kb) [file 13063_2017_2090_MOESM1_ESM.pdf]

[Letterhead: University of Manchester and Salford Together]

Cover letter sent with 1st questionnaire: INTERVENTION

Patient's name and address

Dear [Name of patient],

Care for Older People in Salford: The CLASSIC Study

Six months ago, your GP invited you to participate in a research study being conducted by Salford Royal NHS Trust and the University of Manchester. We want to find out about the care that older people receive for their long-term conditions and how that care affects their health.

Thank you for agreeing to take part in the study and returning '**The Care for Older People in Salford: The CLASSIC Study'** questionnaire. This is the second questionnaire.

We would be grateful if you would complete this questionnaire and return it in the prepaid envelope provided (no stamp is required).

Here is a record of your survey response, which I will update after this survey is completed, and notify you:

Name	Survey 1<br>December 2014	Survey 2<br>July/Aug 2015	Survey 3<br>Jan/Feb 2016
[name of participant]	Completed	………………….	………………..

If for any reason you no longer wish to participate in the study, please send back the blank questionnaire in the envelope provided and we will ensure you receive no further reminders.

If you have any questions about the form please do not hesitate to contact me [Name of manager] by phone xxx xxxx or email: xxx@manchester.ac.uk

All the information you provide will be treated in the strictest confidence.

Many thanks for your help

[Name of manager] Classic study questionnaire manager,
Centre for Primary Care
Health Sciences Research Group - Primary Care
5th Floor Williamson Building
Oxford Road
Manchester M13 9PL

[Letterhead: University of Manchester and Salford Together]

Cover letter sent with 1st questionnaire: CONTROL

Patient's name and address

Dear [Name of patient],

Care for Older People in Salford: The CLASSIC Study

Six months ago, your GP invited you to participate in a research study being conducted by Salford Royal NHS Trust and the University of Manchester. We want to find out about the care that older people receive for their long-term conditions and how that care affects their health.

Thank you for agreeing to take part in the study and returning '**The Care for Older People in Salford: The CLASSIC Study'** questionnaire. This is the second questionnaire.

We would be grateful if you would complete this questionnaire and return it in the prepaid envelope provided (no stamp is required).

If for any reason you no longer wish to participate in the study, please send back the blank questionnaire in the envelope provided and we will ensure you receive no further reminders.

If you have any questions about the form please do not hesitate to contact me [Name of manager] by phone xxx xxxx or email: xxx@manchester.ac.uk

All the information you provide will be treated in the strictest confidence.

Many thanks for your help

[Name of manager] Classic study questionnaire manager,
Centre for Primary Care
Health Sciences Research Group - Primary Care
5th Floor Williamson Building
Oxford Road
Manchester M13 9PL

[Letterhead: University of Manchester and Salford Together]

Cover letter sent with 2nd questionnaire: INTERVENTION (for responders to 1st Questionnaire)

Patient's name and address

Dear [Name of patient],

Care for Older People in Salford: The CLASSIC Study – 12 month follow up

Twelve months ago, your GP invited you to participate in a research study being conducted by Salford Royal NHS Trust and the University of Manchester. We want to find out about the care that older people receive for their long-term conditions and how that care affects their health.

Thank you for your continued participation in the study. It is now time to complete the third questionnaire for the study.

Here is a record of your survey responses:

Name	Survey 1<br>December 2014	Survey 2<br>July/Aug 2015
[name of participant]	Completed	Completed

This questionnaire contains many of the same questions as the last questionnaire, but we would still like you to try to answer all of the questions. The answers you give and the information you provide are important and relevant.

We would be grateful if you would complete this questionnaire and return it in the prepaid envelope provided (no stamp is required).

As a thank you for completing the questionnaire, we will send you a £5.00 high street gift voucher.

If for any reason you no longer wish to participate in the study, please send back the blank questionnaire in the envelope provided and we will ensure you receive no further reminders.

If you have any questions about the form please do not hesitate to contact me [Name of manager] by phone xxx xxxx or email: xxx@manchester.ac.uk

All the information you provide will be treated in the strictest confidence.

Once again, many thanks for your help with this important study.

[Name of manager] – Classic study questionnaire manager
Centre for Primary Care
Health Sciences Research Group - Primary Care
5th Floor Williamson Building
Oxford Road
Manchester M13 9PL

[Letterhead: University of Manchester and Salford Together]

Cover letter sent with 2nd questionnaire: INTERVENTION (for non-responders to 1st Questionnaire)

Patient's name and address

Dear [Name of patient],

Care for Older People in Salford: The CLASSIC Study – 12 month follow up

Twelve months ago, your GP invited you to participate in a research study being conducted by Salford Royal NHS Trust and the University of Manchester. We want to find out about the care that older people receive for their long-term conditions and how that care affects their health.

Thank you for your continued participation in the study. It is now time to complete the third questionnaire for the study.

Here is a record of your survey responses:

Name	Survey 1 December 2014	Survey 2 July/Aug 2015
[name of participant]	Completed	Not completed

This questionnaire contains many of the same questions as the last questionnaire, but we would still like you to try to answer all of the questions. The answers you give and the information you provide are important and relevant.

We would be grateful if you would complete this questionnaire and return it in the prepaid envelope provided (no stamp is required).

As a thank you for completing the questionnaire, we will send you a £5.00 high street gift voucher.

If for any reason you no longer wish to participate in the study, please send back the blank questionnaire in the envelope provided and we will ensure you receive no further reminders.

If you have any questions about the form please do not hesitate to contact me [Name of manager] by phone xxx xxxx or email: xxx@manchester.ac.uk

All the information you provide will be treated in the strictest confidence.

Once again, many thanks for your help with this important study.

[Name of manager] – Classic study questionnaire manager
Centre for Primary Care
Health Sciences Research Group - Primary Care
5th Floor Williamson Building
Oxford Road
Manchester M13 9PL

[Letterhead: University of Manchester and Salford Together]

Cover letter sent with 2nd questionnaire: CONTROL

Patient's name and address

Dear [Name of patient],

Care for Older People in Salford: The CLASSIC Study – 12 month follow up

Twelve months ago, your GP invited you to participate in a research study being conducted by Salford Royal NHS Trust and the University of Manchester. We want to find out about the care that older people receive for their long-term conditions and how that care affects their health.

Thank you for your continued participation in the study. It is now time to complete the third questionnaire for the study.

This questionnaire contains many of the same questions as the last questionnaire, but we would still like you to try to answer all of the questions. The answers you give and the information you provide are important and relevant.

We would be grateful if you would complete this questionnaire and return it in the prepaid envelope provided (no stamp is required).

As a thank you for completing the questionnaire, we will send you a £5.00 high street gift voucher.

If for any reason you no longer wish to participate in the study, please send back the blank questionnaire in the envelope provided and we will ensure you receive no further reminders.

If you have any questions about the form please do not hesitate to contact me [Name of manager] by phone xxx xxxx or email: xxx@manchester.ac.uk

All the information you provide will be treated in the strictest confidence.

Once again, many thanks for your help with this important study.

[Name of manager] – Classic study questionnaire manager
Centre for Primary Care
Health Sciences Research Group - Primary Care
5th Floor Williamson Building
Oxford Road
Manchester M13 9PL
